# Retraction Note: Interplay between cancer cells and M2 macrophages is necessary for miR-550a-3-5p down-regulation-mediated HPV-positive OSCC progression

**DOI:** 10.1186/s13046-021-02112-4

**Published:** 2021-09-30

**Authors:** Ming-xin Cao, Wei-long Zhang, Xiang-hua Yu, Jia-shun Wu, Xin-wei Qiao, Mei-chang Huang, Ke Wang, Jing-biao Wu, Ya-Jie Tang, Jian Jiang, Xin-hua Liang, Ya-ling Tang

**Affiliations:** 1 State Key Laboratory of Oral Diseases & National Clinical Research Center for Oral Diseases, West China Hospital of Stomatology, Sichuan University, No.14, Sec.3, Renminnan Road, Chengdu, 610041 Sichuan China; 2 State Key Laboratory of Microbial Technology, Shandong University, Qingdao, 266237 Shandong China; 3 Department of Head and Neck Surgery, Sichuan Cancer Hospital & Institute, Sichuan Cancer Center, School of Medicine, University of Electronic Science and Technology of China, Chengdu, 610041 Sichuan China

**Retraction Note: J Exp Clin Cancer Res 39, 102 (2020)**

**https://doi.org/10.1186/s13046-020-01602-1**

The Editor-in-Chief has retracted this article due to data duplication in Figs. 2D, 4D and S1F, and remarkable similarities in the backgrounds of the gels presented in Fig. S1A, which have led to a loss of confidence in the presented results and the conclusions drawn in this study.

Author Ya-ling Tang has not explicitly stated whether they agree to this retraction notice. None of the remaining authors have responded to any correspondence from the editor or publisher about this retraction.

